# Distinct Roles of Two Histone Methyltransferases in Transmitting H3K36me3-Based Epigenetic Memory Across Generations in *Caenorhabditis elegans*

**DOI:** 10.1534/genetics.118.301353

**Published:** 2018-09-14

**Authors:** Jeremy Kreher, Teruaki Takasaki, Chad Cockrum, Simone Sidoli, Benjamin A. Garcia, Ole N. Jensen, Susan Strome

**Affiliations:** * Department of Molecular, Cell and Developmental Biology, University of California Santa Cruz, Santa Cruz, California 95064; † Epigenetics Institute, Department of Biochemistry and Biophysics, Perelman School of Medicine, University of Pennsylvania, Philadelphia, Pennsylvania 19104; ‡ Department of Biochemistry and Molecular Biology, University of Southern Denmark, Odense, Denmark 5230

**Keywords:** epigenetics, chromatin, H3K36 methylation, development, germ cells, WormBase

## Abstract

Epigenetic information contributes to proper gene expression and development, and can be transmitted not only through mitotic divisions but also from parents to progeny. We investigated the roles in epigenetic inheritance of MES-4 and MET-1, the two *Caenorhabditis elegans* enzymes that methylate H3K36 (histone H3 Lys 36). Mass spectrometry analysis confirmed immunostaining results showing that both MES-4 and MET-1 catalyze H3K36me3. In the adult germline, MES-4 is enriched in the distal mitotic zone and MET-1 is enriched in the meiotic pachytene zone. Embryos inherit H3K36me3-marked chromosomes from both the oocyte and sperm, and a maternal load of MES-4 and MET-1. Maternal MES-4 quickly associates with sperm chromosomes; that association requires that the sperm chromosomes bear H3K36me3, suggesting that MES-4 is recruited to chromosomes by preexisting H3K36me3. In embryos that inherit H3K36me3-positive oocyte chromosomes and H3K36me3-negative sperm chromosomes, MES-4 and H3K36me3 are maintained on only a subset of chromosomes until at least the 32-cell stage, likely because MES-4 propagates H3K36me3 on regions of the genome with preexisting H3K36me3. In embryos lacking MES-4, H3K36me3 levels on chromosomes drop precipitously postfertilization. In contrast to the relatively high levels of MES-4 in early-stage embryos, MET-1 levels are low at early stages and start increasing by the ∼26-cell stage, consistent with expression from the zygotic genome. Our findings support the model that MET-1 mediates transcription-coupled H3K36me3 in the parental germline and transcriptionally active embryos, and that MES-4 transmits an epigenetic memory of H3K36me3 across generations and through early embryo cell divisions by maintaining inherited patterns of H3K36me3.

MULTICELLULAR organisms must generate a wide array of cell types from a single cell, the zygote, and must ensure that cell fates are maintained during the lifetime of the organism. Failure to do either can lead to lethality, developmental defects, and cancer. Establishment and maintenance of different cell fates relies on a variety of mechanisms to generate different gene expression patterns between cells that possess an identical genome sequence. One mechanism is packaging sets of genes into chromatin states that are more or less accessible to the transcriptional machinery. The first level of DNA packaging into chromatin entails wrapping DNA around octamers of histone proteins (Kornberg 1974; Olins and Olins 1974). Further levels of packaging occur in response to numerous factors, including covalent modifications on histone tails. Histone tail modifications can influence chromatin by modulating DNA–histone interactions or via proteins that bind to those modifications (*e.g.*, Deuring *et al.* 2000; Corona *et al.* 2002; Carrozza *et al.* 2005). Diverse combinations of histone tail modifications provide the potential for gene regulatory information to be encoded in the chromatin fiber (Jenuwein and Allis 2001; Bannister and Kouzarides 2011).

Actively expressed genes are typically packaged with nucleosomes containing histone H3 trimethylated at Lys 36 (H3K36me3) (Rao *et al.* 2005; Furuhashi *et al.* 2010; Rechtsteiner *et al.* 2010), while repressed genes are often packaged with nucleosomes containing histone H3 trimethylated at Lys 27 (H3K27me3) (Kirmizis *et al.* 2004; Lee *et al.* 2006; Tolhuis *et al.* 2006). Studies of H3K27me3 have established several important paradigms, as summarized here. An involvement of H3K27me3 in gene repression was discovered in *Drosophila* (Müller *et al.* 2002). During *Drosophila* embryogenesis, transiently expressed transcription factors dictate which *Hox* genes are expressed and which are repressed in each body segment. A memory of *Hox* gene repression is maintained through development by Polycomb Repressive Complexes 1 and 2 (PRC1 and PRC2) (Schuettengruber *et al.* 2007). PRC2 mediates repression via methylation of H3K27 by E(z) (Müller *et al.* 2002). The repressive role of E(z)/PRC2 and H3K27me3 is conserved across many species, including *Caenorhabditis elegans*, *Drosophila*, mammals, and plants (Cao *et al.* 2002; Kuzmichev *et al.* 2002; Bender *et al.* 2004; Lindroth *et al.* 2004; Steffen and Ringrose 2014). A critical question is how H3K27me3 marking and repression are maintained through DNA replication and cell division, given the eviction of nucleosomes that occurs in advance of DNA polymerase. A current well-supported model is that: (1) evicted parental H3/H4 histones are held near the replication fork and incorporated randomly into the two daughter chromatids (Margueron and Reinberg 2010; Lanzuolo *et al.* 2011), and this passes H3K27me3-marked histones to daughter chromatids; (2) new histones are incorporated into daughter chromatids to restore nucleosome density; and (3) H3K27me3-marked parental histones recruit PRC2 and stimulate its histone methyltransferase (HMT) activity to restore H3K27me3 to high levels on daughter chromatids (Margueron *et al.* 2009; Poepsel *et al.* 2018).

This report focuses on the generation and maintenance of H3K36me3, which is less well understood than H3K27me3. H3K36me3 is conserved from yeast to humans and is generally associated with actively expressed genes (Sun *et al.* 2005; Barski *et al.* 2007; Wagner and Carpenter 2012). The paradigm that H3K36 methylation is deposited cotranscriptionally came from budding yeast, in which a single enzyme, Set2, generates all three levels of methylation (me1, me2, and me3) (Strahl *et al.* 2002). Set2 has a Set2 Rpb1 Interacting (SRI) domain, through which it associates with the C-terminal domain of RNA Polymerase II to deposit H3K36me in the body of genes during transcription elongation (Strahl *et al.* 2002; Kizer *et al.* 2005). The discovery that multicellular organisms have multiple H3K36 HMTs (*e.g.*, two in *C. elegans*, two in *Drosophila*, and at least four in mammals) (Bender *et al.* 2006; Bell *et al.* 2007; Edmunds *et al.* 2008; Li *et al.* 2009; Furuhashi *et al.* 2010; Rechtsteiner *et al.* 2010; Wagner and Carpenter 2012) raises the question of whether there has been diversification of the activities and functions of H3K36 HMTs (Wagner and Carpenter 2012; McDaniel and Strahl 2017). In these organisms, different HMTs are thought to be devoted to generating either H3K36me2 or H3K36me3 (Wagner and Carpenter 2012). Furthermore, our previous studies suggest that the two *C. elegans* H3K36 HMTs serve different functions. MET-1, like yeast Set2, likely generates H3K36me in a cotranscriptional manner, while MES-4 can maintain H3K36me in a manner that does not require ongoing transcription; specifically, in *C. elegans* embryos, based on chromatin immunoprecipitation analysis, maternally provided MES-4 maintains H3K36me on genes that were expressed in the parental germline regardless of whether those genes are transcribed in embryos (Furuhashi *et al.* 2010; Rechtsteiner *et al.* 2010). Although MES-4 is not required in adults for germline maintenance and function, absence of maternal MES-4 from embryos causes the nascent germ cells to die during larval development (Capowski *et al.* 1991; Garvin *et al.* 1998). These findings support the following model: (1) in parental germ cells, MET-1 deposits H3K36me on expressed genes during transcription; (2) in embryos, MES-4 maintains H3K36me on those genes; and (3) delivery of chromosomes with H3K36me marking of germline-expressed genes to the primordial germ cells (PGCs) enables those cells to launch a proper germline transcription program.

In this study, we addressed questions raised by the model of MET-1 and MES-4 action in *C. elegans*, focusing on H3K36me3. We show that both MET-1 and MES-4 contribute to H3K36me3. To our knowledge, this is the first example of two different HMTs contributing to H3K36me3 (Wagner and Carpenter 2012). The two HMTs differ in their temporal and spatial expression patterns in germlines and embryos, as well as their chromosomal targets; during germline development, H3K36me3 marking of the autosomes is accomplished by both MET-1 and MES-4, while H3K36me3 marking of the X chromosomes during oogenesis is accomplished by MET-1. Both enzymes are maternally transmitted to the embryo at fertilization. Maternal MES-4 associates with sperm chromosomes soon after fertilization, and that association requires that the sperm chromosomes arrive already marked with H3K36me3. During the early embryonic cleavages, MES-4 levels stay high and MES-4 is responsible for maintaining inherited patterns of H3K36me3. In contrast, MET-1 levels rapidly diminish after fertilization and increase coincident with zygotic genome activation. These findings support MET-1 marking genes cotranscriptionally and MES-4 serving a transgenerational epigenetic role to maintain gene expression information transmitted from parent germ cells to the PGCs in progeny.

## Materials and Methods

### Strains and culture

*C. elegans* were maintained at 15 or 20° on NGM (Nematode Growth Medium) agar plates using *Escherichia coli* OP50 as a food source. Experiments were carried out at 20, 24, 25, 25.5, or 26.5°. Strains used for this study include:

N2 (Bristol) as wild-type.
DH0245 *fem-2*(*b245ts*) *III*.
SS0875 *met-1*(*n4337*) *I/hT2-GFP* (*I*;*III*); *mes-4*(*bn73*) *dpy-11*(*e224*) *V/DnT1*[*unc*(*n754*) *let*] (*IV*;*V*).
SS1095 *mes-4*(*bn73*) *V/DnT1-GFP*[*unc*(*n754*) *let qIs51*] (*IV*;*V*).
SS1139 *met-1*(*tm1738*) *I/hT2-GFP* (*I*;*III*).
SS1140 *met-1*(*n4337*) *I/hT2-GFP* (*I*;*III*).
SS1278 *jmjd-2*(*tm2966*) *II*; *jhdm-1*(*tm2819*) *III*; *mes-4*(*bn73*) *V/DnT1*[*unc*(*n754*) *let*] (*IV*;*V*).

The longest *met-1* ORF includes 14 exons. *met-1*(*tm1738*) deletes 656 bp that extend from the last AG of intron 3 to almost the end of exon 5 (I: 4,255,715–4,256,370), and *met-1*(*n4337*) deletes 1860 bp that extend from almost the end of exon 5 to the first GT of intron 8 (I: 4,256,309–4,258,168). Using an anti-MET-1 antibody directed against amino acids 1263–1362 (which span exons 12 and 13; described below), *met-1*(*tm1738*) eliminates MET-1 staining, *met-1*(*n4337*) does not.

### Histone extraction

The histone extraction protocol was adapted from Lin and Garcia (2012). Worms were grown in liquid culture and embryos were collected by digesting adults with an alkaline-bleach solution (1% NaOCl in 0.5 M NaOH). Embryos were frozen in liquid nitrogen for storage. Embryo populations were staged by fixing a sample of collected embryos with methanol before freezing and imaging nuclei stained with DAPI. Populations of early embryos were between 62 and 92% < 100-cell. Frozen wild-type and *met-1* mutant early embryos were thawed in 10 ml modified nuclei purification buffer (NPB) (10 mM Tris pH 7.5, 40 mM NaCl, 90 mM KCl, 2 mM EDTA, 0.5 mM EGTA, 1 mM DTT, 0.5 mM spermidine, 0.25 mM spermine, 0.1% Triton X-100, Roche EDTA-free protease inhibitor cocktail, 10 mM sodium butyrate, and 10 mM glycerolphosphate), then homogenized with a glass dounce homogenizer and 30 strokes of a tight-fitting pestle to free nuclei. Nuclei were enriched by pelleting cellular debris at 100 × *g* for 2 min at 4°, collecting the supernatant, adjusting the volume to 45 ml with modified NPB, pelleting residual debris at 100 × *g* for 5 min, and collecting the supernatant. Enriched nuclei were washed twice in modified NPB by centrifuging at 1000 × *g* for 10 min. Nuclei were resuspended in 400 µl 0.4 M NH_2_SO_4_, vortexed briefly to ensure nuclei were completely resuspended, and rotated overnight at 4°. Samples were centrifuged at 16,000 × *g* for 10 min at 4° to pellet insoluble debris. The supernatants containing histones were transferred to clean tubes and histones were precipitated by the addition of 100% trichloroacetic acid to a final concentration of 33%. Samples were rotated overnight at 4°, then centrifuged at 16,000 × *g* for 10 min at 4°. The pellets containing histones were washed twice with 1 ml ice-cold acetone. After the second wash, the pellets were allowed to air dry for 20 min at room temperature.

### Mass spectrometry

Histone propionylation and digestion were performed as previously described with minor modification (Sidoli *et al.* 2016). Propionic anhydride solution was freshly prepared by mixing propionic anhydride with 2-propanol in a ratio of 1:3 (v/v), creating the propionylation mix. Next, 15 μl of propionylation mix was added to the histone sample in the ratio of 1:2 (v/v), immediately followed by 7.5 μl of NH_4_OH to adjust the pH to ∼8.0. Samples were incubated for 15 min at 37°. Propionylation was repeated a second time after drying samples in a SpeedVac centrifuge. Samples were dried and resuspended in 50 mM NH_4_HCO_3_ overnight at room temperature with trypsin at an enzyme:sample ratio of 1:20. After digestion, the derivatization reaction was performed again twice to derivatize the N-termini of peptides. Samples were desalted using C_18_ Stage-tips before LC-MS (liquid chromatography-mass spectrometry) analysis. Samples were analyzed using a nanoLC-MS/MS setup. First, 1 µg of sample was loaded onto an in-house packed 75-μm ID × 20 cm Reprosil-Pur C18-AQ (3 μm; Dr. Maisch HPLC GmbH, Ammerbuch, Germany) nano-column using an EASY-nLC nano-HPLC (Thermo Scientific, San Jose, CA). Peptides were separated with a linear gradient using two buffers: A = 0.1% formic acid, and B = 95% acetonitrile and 0.1% formic acid. Elution of histone peptides was achieved using a gradient of 0–26% buffer B over 45 min; then the column was washed by running from 26 to 80% buffer B over 5 min followed by isocratic 80% buffer B for 10 min. The flow rate was set at 300 nl/min. Nano-liquid chromatography (nLC) was coupled online with an Orbitrap Elite MS (Thermo Scientific). Runs were acquired using data-independent acquisition (DIA) as described (Sidoli *et al.* 2015). Briefly, two full-scan MS spectra (*m/z* 300−1100) were acquired in the Orbitrap at a resolution of 120,000 (at 200 *m/z* full width at half maximum) in between 16 MS/MS events spanning the *m/z* range, each acquired in the ion trap with an isolation window of 50 *m/z*. Fragmentation was performed by using collision-induced dissociation set at 35%. Raw MS data were analyzed using Skyline (MacLean *et al.* 2010) by performing extracted ion chromatography of the different modified and unmodified isoforms of the H3 peptide KSAPTTGGVKKPHR (amino acids 27–40). MS/MS chromatographic profiles, acquired by DIA, were used to increase the confidence in the correct signal to extract. The relative abundance of post-translational modifications was determined by dividing the area of a particular isoform by the summed total area of all peptide isoforms.

### Immunostaining

The immunostaining protocol was adapted from Strome and Wood (1983). Gravid adult worms were dissected to isolate germlines, oocytes, and embryos. Dissections were done in drops of Egg Buffer (25 mM HEPES pH 7.4, 118 mM NaCl, 48 mM KCl, 2 mM EDTA, and 5 mM EGTA) on a polylysine-coated slide. After dissection, a coverslip was placed over the sample and the slide was immersed in liquid nitrogen for at least 2 min. The coverslip was removed and the samples were fixed in methanol at 4° for 10 min, followed by acetone at 4° for 10 min, and then air dried. Slides were incubated with 1.5% ovalbumin/1.5% bovine serum albumin in PBS-T (1× PBS and 0.1% Tween 20) for 30 min at room temperature, followed by primary antibody diluted in PBS-T overnight at 4°. Primary antibody dilutions were: 1:50,000 mouse monoclonal anti-H3K36me3 (gift from Hiroshi Kimura), 1:20,000 rabbit anti-MET-1 (generated against amino acids 1263–1362 by Strategic Diagnostics, Newark, DE), and 1:500 rabbit anti-MES-4 (generated against the C-terminal 19 amino acids + Cys and affinity purified; Bender *et al.* 2006). Slides were washed three times for 10 min each in PBS-T at room temperature, and then incubated with 1:300 Alexa Fluor secondary antibodies (Life Technologies) diluted in PBS-T for 2 hr at room temperature. Slides were washed three times for 10 min each in PBS-T at room temperature and mounted in Gelutol mounting fluid.

**Figure 1:**
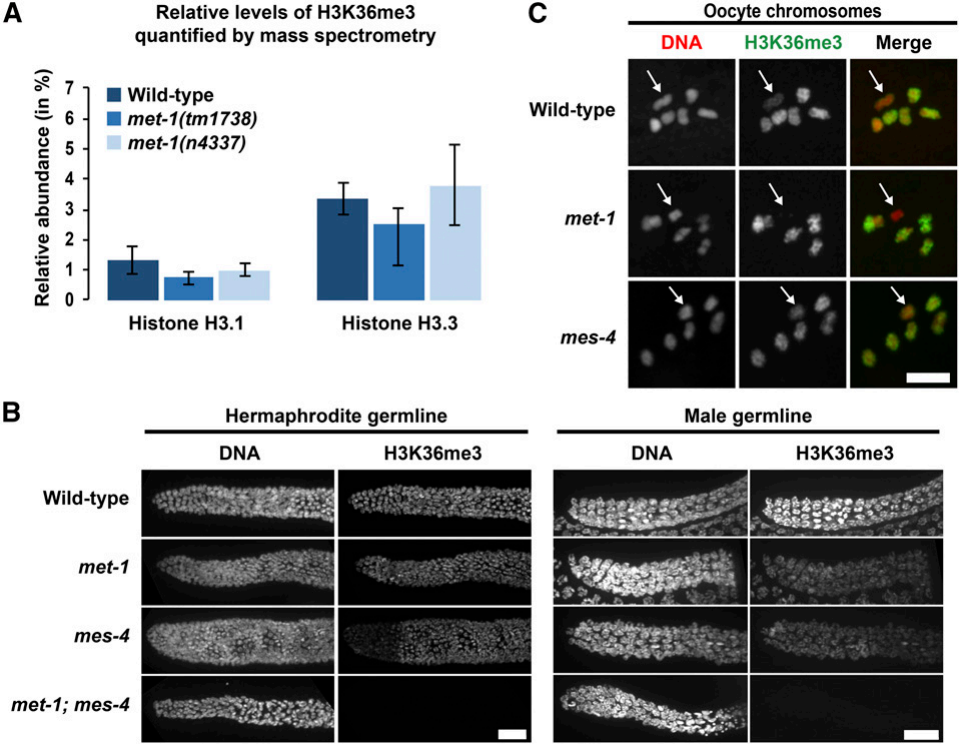
H3K36me3 is generated by both MET-1 and MES-4 in germlines and embryos, but MET-1 is solely responsible for H3K36me3 on the oocyte X chromosome. (A) Relative abundance of H3.1K36me3 and H3.3K36me3 peptide fragments from wild-type, *met-1*(*tm1738*), and *met-1*(*n4337*) embryonic nuclei, as determined by mass spectrometry. Percent abundance is relative to total H3.1 or H3.3 peptides detected. Error bars represent SEM. (B) Immunofluorescence images showing DNA and H3K36me3 in distal germlines from wild-type, *met-1*(*n4337*), *mes-4*(*bn73*), and *met-1*(*n4337*); *mes-4*(*bn73*) adult hermaphrodites and males. For each genotype, 8–12 hermaphrodite gonads and 5–14 male gonads were analyzed. All samples analyzed supported reduction of H3K36me3 in the single mutants and absence of detectable H3K36me3 in double mutants. Six of 12 *mes-4* hermaphrodite germlines displayed reduced H3K36me3 signal in the distal region (left side of image), as shown in (B); the other six appeared more uniform. H3K36me3 in all eight of the *mes-4* male germlines appeared uniform, as shown in (B). Bar, 20 µm. (C) Immunofluorescence images showing DNA (red) and H3K36me3 (green) on oocyte chromosomes from wild-type, *met-1*(*n4337*), and *mes-4*(*bn73*) hermaphrodites. White arrow indicates the presumed X chromosome bivalent, based on lower or undetectable H3K36me3 staining compared to the autosomes. For each genotype, 9–12 oocytes were analyzed. Bar, 5 µm.

Some images were acquired with a Volocity spinning-disk confocal system (Perkin-Elmer/Improvision, Norwalk, CT) fitted on a Nikon Eclipse TE2000-E inverted microscope (Garden City, NY) [Figure 1B (male germlines), Figure 3B, and Figure 7]. All other images were acquired using a Solamere spinning-disk confocal system controlled by µManager software (Edelstein *et al.* 2014). The set-up was as follows: Yokogawa CSUX-1 scan head, Nikon Eclipse TE2000-E inverted microscope, Hamamatsu ImageEM 32 camera, Plan Apo 63×/1.4 numerical aperture oil objective, and Plan Apo 100×/1.4 numerical aperture oil objective. Germlines shown in Figure 1B and Figure 5A were straightened postacquisition using the ImageJ straighten plugin (Schneider *et al.* 2012).

### Quantification of immunostaining

The quantification protocol used for Figure 4B was adapted from McCloy *et al.* (2014) and Burgess *et al.* (2010). Images were acquired using the Solamere imaging set-up described above and used for quantification of H3K36me3 antibody staining, which was performed using the protocol and antibodies described above. All images were acquired within the linear range and analyzed in ImageJ. In brief, a region was drawn around chromosomes using the DAPI channel, then the integrated density (intensity) of the H3K36me3 signal within the region was measured. Background signal was determined by measuring the intensity of three circular spots outside of the nucleus and averaging their intensity. The background-normalized intensity measurements presented in Figure 4B were calculated by measuring the H3K36me3 signal intensity within a region of interest and then subtracting the average background intensity multiplied by the area of the region of interest.

### Analysis of fertility

To score fertility, wild-type and *met-1* heterozygous L4s were shifted from 20° to the experimental temperature (25, 25.5, or 26.5°). Their wild-type and *met-1* homozygous L4 F1 progeny were picked to new plates, then scored for fertility ∼24-hr later. Selection of L4s for scoring prevented biased selection of fertile or sterile worms. Worms were visually scored using a Leica M80 stereo microscope. Worms containing embryos were scored as fertile and worms lacking embryos were scored as sterile. Worms that were not obviously fertile or sterile were picked onto individual plates, and scored as fertile if they laid embryos and sterile if they did not. Fertile wild-type and homozygous *met-1* mutants were chosen from each generation to produce progeny to score in the next generation.

### RNA interference depletion of CSR-1

Wild-type hermaphrodites were fed bacteria expressing double-stranded RNA against *csr-1* [from the Ahringer RNA interference (RNAi) feeding library (Kamath and Ahringer 2003)]. To generate *csr-1*(*RNAi*) embryos, hermaphrodites were placed on RNAi feeding plates as synchronized L1s and cultured at 24° until they started producing embryos.

### Data availability

MS raw data are available at https://chorusproject.org, project number 1495. All strains and noncommercially available reagents are available upon request.

## Results

### In C. elegans, H3K36me3 is generated by both MET-1 and MES-4

MET-1-related HMTs in other organisms, Set2 in *Drosophila* and SETD2 in mammals, are thought to be fully responsible for H3K36me3 (Bell *et al.* 2007; Edmunds *et al.* 2008; Wagner and Carpenter 2012). However, previous immunostaining results suggested that in *C. elegans*, both MET-1 and MES-4 contribute to H3K36me3: the level of H3K36me3 immunostaining in embryos is high in wild-type, reduced in *met-1* mutants, reduced in *mes-4* mutants, and undetectable in double *met-1*; *mes-4* mutants (Furuhashi *et al.* 2010; Rechtsteiner *et al.* 2010). To test by an antibody-independent method if an enzyme other than MET-1 contributes to H3K36me3, we performed mass spectrometry (MS) analysis of H3 tails from wild-type and *met-1* mutant early embryos. Embryos bearing either of two deletion alleles of *met-1* (see *Materials and Methods* for allele descriptions) had robust levels of H3K36me3, which must be generated by a different HMT (Figure 1A). MES-4 is the only other candidate H3K36me3 HMT identified in *C. elegans* to date. We could not analyze *mes-4* mutant embryos or *mes-4*; *met-1* double-mutant embryos, because the maternal-effect sterility of those strains prevented us from collecting sufficient quantities of those mutant embryos for MS. In combination with immunostaining analysis of embryos (Furuhashi *et al.* 2010; Rechtsteiner *et al.* 2010) and germlines (Figure 1B), in which H3K36me3 is present in *met-1* mutants but not detectable in *met-1*; *mes-4* double mutants, our MS results support MES-4 contributing to H3K36me3. Our MS results further indicate that MES-4 catalyzes H3K36 methylation on both canonical H3 protein (H3.1 in *C. elegans*) and the H3 variant H3.3. Typically, canonical H3 is expressed and deposited exclusively during S phase (replication-dependent), while H3.3 is expressed throughout the cell cycle and is deposited at regions of active transcription (replication-independent) (Talbert and Henikoff 2017).

### In germ cells, both MET-1 and MES-4 generate H3K36me3 on the autosomes at all stages, and MET-1 additionally generates H3K36me3 on the X chromosomes in late oogenesis

To determine the spatial and temporal pattern of H3K36me3 during germ cell development, we analyzed the distribution of H3K36me3 in germlines and gametes. Immunostaining of dissected germlines revealed chromosome-associated H3K36me3 signal in all germ nuclei, including mitotic and meiotic germ cells, and mature oocytes (Figure 1, B and C). Consistent with previous findings that the X chromosomes are transcriptionally repressed in the germline (Reinke *et al.* 2000, 2004; Kelly *et al.* 2002), H3K36me3 staining was observed on the autosomes but not on the X chromosomes from the distal end of the mitotic zone through late pachytene (data not shown). In contrast, all six bivalents in the oocyte, including the X bivalent, stained positively for H3K36me3 (Figure 1C); this is consistent with previously documented turn-on of X-linked genes at late stages of oogenesis (Kelly *et al.* 2002).

To investigate the spatial activity of MES-4 and MET-1 in the germline, we immunostained dissected germlines from *met-1* and *mes-4* mutant hermaphrodites. H3K36me3 was detected in *met-1* mutant germlines, indicating that MES-4 generates H3K36me3 throughout the germline and in oocytes (Figure 1, B and C). H3K36me3 was also detected in *mes-4* mutant germlines, indicating that MET-1 generates H3K36me3 throughout the germline and in oocytes (Figure 1, B and C). H3K36me3 staining of the X bivalent in oocytes was detected in *mes-4* mutant germlines but not in *met-1* mutant germlines (Figure 1C), indicating that H3K36me3 on the X is generated by MET-1. These results show that MES-4 and MET-1 each generate H3K36me3 at all germline stages, and suggest that the X chromosomes are uniquely methylated during late oogenesis by MET-1. Although MES-4 apparently does not *de novo* methylate the X chromosomes in *met-1* mutant germlines, it may maintain MET-1-generated H3K36me3 on the X chromosomes in wild-type germlines.

### met-1 mutants display low-level sterility at elevated temperature

**Figure 2:**
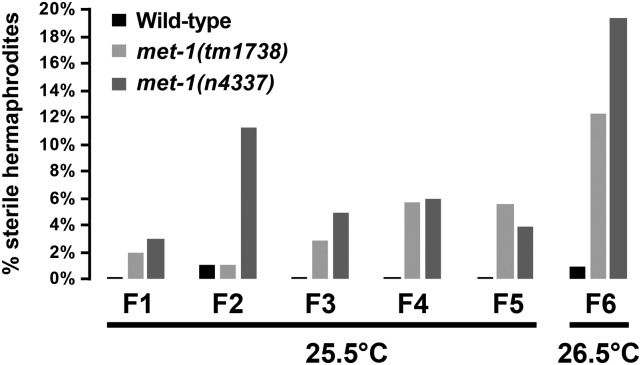
*met-1* mutants display low-level sterility at elevated temperatures. The percentage of sterile hermaphrodites in each generation is shown for wild-type and *met-1* mutants. Hermaphrodites were scored as fertile *vs.* sterile on a dissecting microscope based on presence or absence of embryos in their uterus. The number of hermaphrodites scored was ≥ 100 for all generations and genotypes except F2 *met-1*(*tm1738*) was 95, F6 *met-1*(*tm1738*) was 81, and F6 *met-1*(*n4337*) was 98.

*mes-4* mutants have a strict maternal-effect sterile phenotype at all temperatures (Capowski *et al.* 1991), while *met-1* mutants do not display sterility at the standard laboratory temperature of 20° (Andersen and Horvitz 2007). Because the mutant phenotypes of many germline-active genes are enhanced at elevated temperature (*e.g.*, Kawasaki *et al.* 2004; Spike *et al.* 2008), we tested *met-1* mutants over several generations of growth at 25 and 25.5°; these temperatures are just below the temperature (26°) at which some wild-type worms develop into sterile adults. Both *met-1* alleles caused a small percentage of worms to develop into sterile adults at 25°. Even when cultured at 25° for three generations, only 0.9% of *met-1*(*tm1738*) and 3.1% of *met-1*(*n4337*) were sterile. In a second experiment, we observed a slightly higher level of sterility for *met-1*(*tm1738*) and *met-1*(*n4337*) at 25.5° after five generations, 5.6 and 4.0%, respectively (Figure 2). We challenged the mutants further by shifting generation 6 L4s to 26.5°. That caused an increase in percent sterility not observed in wild-type worms (Figure 2). Thus, *met-1* mutants are generally fertile at all temperatures, but at elevated temperature are more likely than wild-type worms to develop into sterile adults.

### H3K36me3-marked chromosomes are transmitted to embryos by both sperm and oocytes

**Figure 3:**
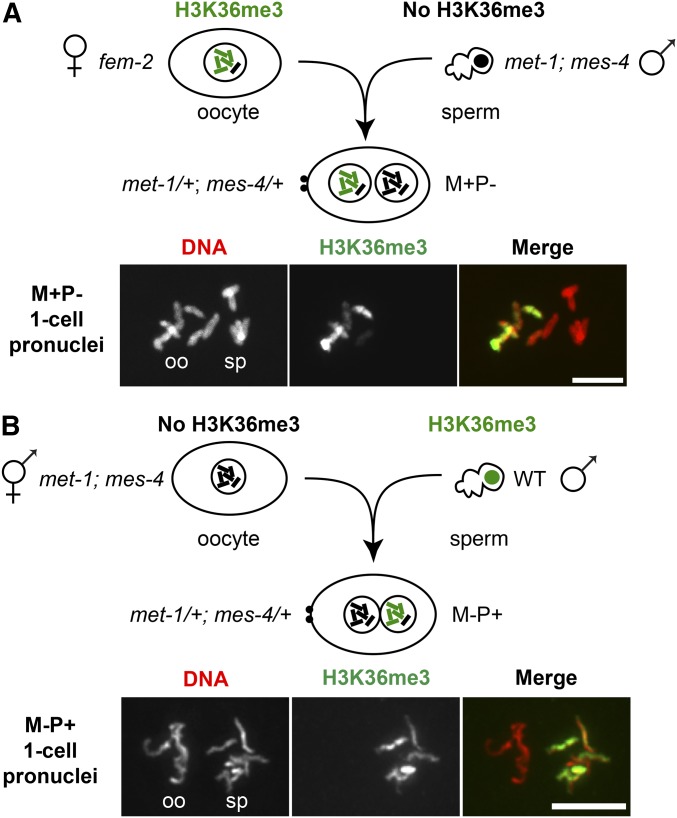
Oocytes and sperm transmit H3K36me3-marked chromosomes to the embryo. (A) Immunofluorescence images of DNA (red) and H3K36me3 (green) staining of prometaphase chromosomes in a one-cell M+P− embryo produced by a *fem-2* female mated with a *met-1*(*n4337*); *mes-4*(*bn73*) male. Oocyte- (oo) and sperm-derived (sp) chromosomes were identified by their position relative to the polar bodies (data not shown). (B) Immunofluorescence images of DNA (red) and H3K36me3 (green) staining of prometaphase chromosomes in a one-cell M-P+ embryo produced by a *met-1*(*n4337*); *mes-4*(*bn73*) hermaphrodite mated with a wild-type (WT) male. Maternally supplied chromosomes (M) or paternally supplied chromosomes (P) with H3K36me3 (+) or lacking H3K36me3 (−). Bar, 5 µm.

The fertility defects observed in *met-1* and especially *mes-4* mutants suggest that marking of chromatin by H3K36me3 is important for germline function and propagation of the species. We demonstrated above that both MET-1 and MES-4 contribute to generating H3K36me3 in the parental germline, and on the chromosomes that are packaged into oocytes (Figure 1C). To test each gamete’s contribution of H3K36me3-marked chromosomes to embryos, we mated parents that were capable of generating H3K36me3 with *met-1*; *mes-4* parents that were incapable of generating H3K36me3, and immunostained the resulting one-cell embryos. In one-cell embryos in which H3K36me3 transmission was possible by the maternal (M) gamete but not the paternal (P) gamete (so M+P− embryos), the oocyte-delivered chromosomes were H3K36me3 positive and the sperm-delivered chromosomes were H3K36me3 negative (Figure 3A). Conversely, in one-cell embryos in which H3K36me3 transmission was possible by the paternal gamete but not the maternal gamete (so M−P+ embryos), the oocyte-delivered chromosomes were H3K36me3 negative and the sperm-delivered chromosomes were H3K36me3 positive (Figure 3B). These findings reveal that both oocytes and sperm transmit H3K36me3-marked chromosomes from the parental germline to the one-cell embryo.

### Maternal MES-4 ensures that H3K36me3 marking in embryos persists beyond the four-cell stage

**Figure 4:**
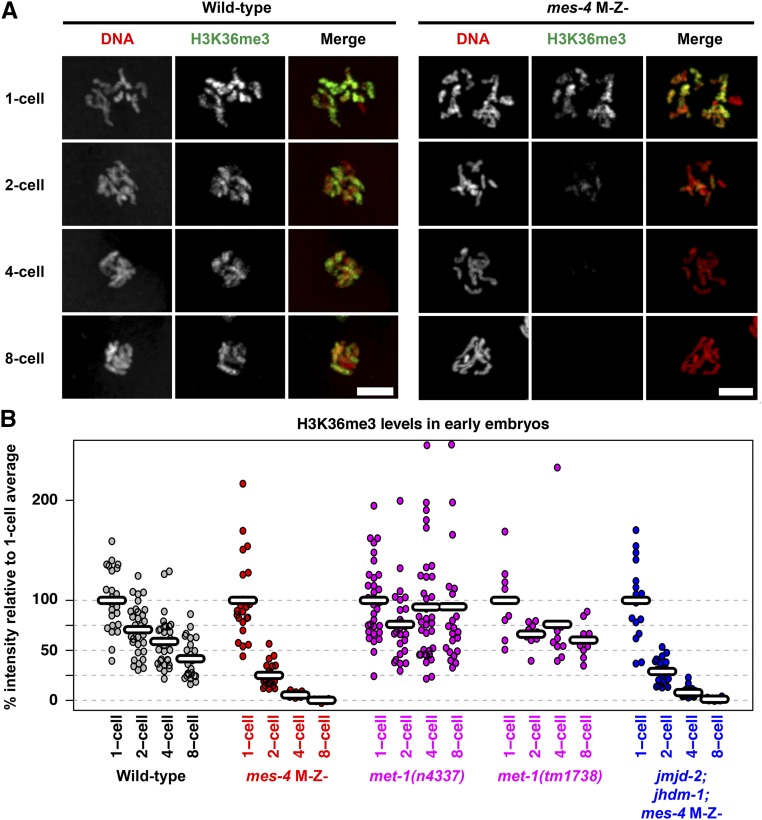
MES-4, but not MET-1, maintains H3K36me3 through cell divisions in early embryos. (A) Immunofluorescence images of DNA (red) and H3K36me3 (green) in nuclei from wild-type and M-Z- *mes-4*(*bn73*) embryos. Bar, 5 µm. (B) Quantification of nuclear H3K36me3 immunofluorescence intensity in single nuclei of wild-type, *mes-4*(*bn73*) mutant, *met-1* mutant, and *jmjd-2*(*tm2966*); *jhdm-1*(*tm2918*); *mes-4*(*bn73*) triple-mutant embryos. For each genotype, percent intensity is relative to the average intensity of the two juxtaposed pronuclei in one-cell embryos, which was set to 100%. Each point represents an individual nucleus. Horizontal marks represent the mean. The two upper-most points in *met-1*(*n4337*) four- and eight-cell samples were 293 and 399%, respectively, but were placed within the scale shown to display details for the majority of data points. M, maternal supply of gene product; Z, zygotic synthesis of gene product.

We previously reported that MET-1 is likely a transcription-coupled H3K36 HMT capable of *de novo* methylation and that MES-4 is a transcription-independent H3K36 HMT devoted to maintenance of that mark (Bender *et al.* 2006; Furuhashi *et al.* 2010; Rechtsteiner *et al.* 2010). Since the cells in early *C. elegans* embryos are largely transcriptionally silent (Seydoux and Dunn 1997; Baugh *et al.* 2003; Boeck *et al.* 2016), we predicted that MES-4 and not MET-1 would be critical for maintaining H3K36me3 in early embryos. To test that prediction, we generated embryos that inherited H3K36me3-marked chromosomes, but either no MES-4 or no MET-1, and analyzed levels of chromosomal H3K36me3 at progressively later stages of embryogenesis. To eliminate MES-4, we used *mes-4* M-Z- mutant embryos that lack maternally loaded MES-4 (M-) and are unable to produce zygotic MES-4 (Z-). These embryos inherited MET-1-generated H3K36me3. We quantified the intensity of H3K36me3 staining during prometaphase in one-cell- to eight-cell-stage embryos. For each genotype, we compared the average intensity in single diploid nuclei of two-, four-, and eight-cell embryos to the average intensity in the two juxtaposed haploid pronuclei in one-cell embryos. Staining of wild-type embryos revealed that the average intensity decreased from the one-cell stage (set to 100%) to the two-cell (71%), four-cell (59%), and eight-cell (42%) stages (Figure 4, A and B). In *mes-4* M-Z- embryos that lacked MES-4, the decrease was more rapid, dropping from the one-cell stage (set to 100%) to the two-cell (25%) and four-cell (5%) stages; staining was undetectable by the eight-cell stage (0%) (Figure 4, A and B). In contrast, *met-1* mutant embryos had H3K36me3 levels that were similar to or slightly higher than wild-type controls (Figure 4B). Therefore, MES-4 but not MET-1 is required to maintain wild-type levels of H3K36me3 through the early embryonic divisions and to ensure that H3K36me3 marking persists beyond the four-cell stage. We expect that this is due to MES-4-mediated methylation of H3K36; another possibility is that MES-4 influences the rate of histone exchange.

We noted that the rate of H3K36me3 loss in *mes-4* M-Z- embryos was greater than expected if the parental load of H3K36me3 was simply being diluted by rounds of DNA replication. Loss of H3K36me3 by dilution at each round of DNA replication would predict a drop of 50% at each subsequent stage, *i.e.*, 100% at the one-cell stage, then 50, 25, and 12.5% in each nucleus at the two-, four-, and eight-cell stages, respectively. Even when we adjusted for the decrease seen in wild-type worms, the rate of loss in *mes-4* mutants was still greater than the decrease expected by simple dilution, leading us to consider the possibility that H3K36me3 removal is an active process involving demethylation. Two different demethylases have been reported to target H3K36me3 in *C. elegans*: JMJD-2 and JHDM-1(Tsukada *et al.* 2006; Whetstine *et al.* 2006). If JMJD-2, JHDM-1, or both demethylate H3K36 in early embryos, we predicted that H3K36me3 levels would be higher in *jmjd-2*; *jhdm-1*; *mes-4* M-Z- embryos than in *mes-4* M-Z- embryos. We did not observe a difference between H3K36me3 levels in *jmjd-2*; *jhdm-1*; *mes-4* M-Z- embryos compared to *mes-4* M-Z- embryos (Figure 4B). These results suggest that the dramatic drop in H3K36me3 in *mes-4* mutant embryos is not due to demethylation by JMJD-2 or JHDM-1. Another demethylase(s) may be involved or histone exchange may deplete H3K36me3.

### MET-1 and MES-4 are maternally supplied to embryos, and MES-4 is the major H3K36me3 HMT in early embryos

**Figure 5:**
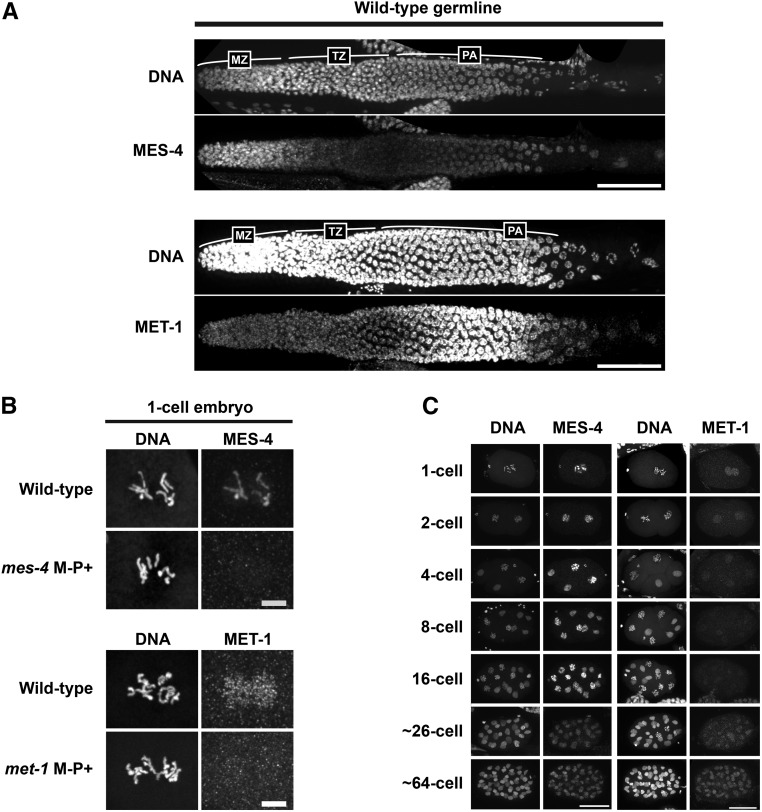
MET-1 and MES-4 have different spatial and temporal protein accumulation patterns in germlines and embryos. (A) Immunofluorescence images showing DNA, and MET-1 or MES-4, in wild-type hermaphrodite germlines. Germline images are oriented with the distal tip to the left and late pachytene to the right. MZ, mitotic zone; TZ transition zone; PA, pachytene. Bar, 50 µm. (B) Immunofluorescence images of one-cell embryos showing DNA and the maternal contribution of MES-4 or MET-1 in wild-type embryos. The paternal contribution was assessed in M-P+ embryos generated by mating *mes-4*(*bn73*) females or *met-1*(*tm1738*) females (M−) with wild-type males (P+). Bar, 5 µm. (C) Immunofluorescence images showing DNA, and MES-4 or MET-1, in wild-type embryos. Bar, 20 µm.

Immunostaining wild-type germlines for MES-4 and MET-1 revealed different protein accumulation patterns. MES-4 is enriched in the distal mitotic region and the late pachytene region (Fong *et al.* 2002; Figure 5A), while MET-1 is low in the distal mitotic region, increases in the midpachytene region, and drops again during later pachytene (Figure 5A). To determine if MES-4 and MET-1 proteins are transmitted from the germline to embryos through the oocyte, the sperm, or both gametes, we stained one-cell embryos. We detected immunostaining of both proteins in one-cell embryos from wild-type hermaphrodites. MES-4 staining was mainly chromosomally associated, while MET-1 staining was nucleoplasmic and low-level (Figure 5B). One-cell embryos from *mes-4* or *met-1* mutant mothers mated to wild-type males lacked detectable staining of the respective protein (Figure 5B). These results demonstrate that all detectable MES-4 and MET-1 present in one-cell embryos is maternally supplied via the oocyte or translated from maternal transcripts. The results further demonstrate that MES-4 association with sperm chromosomes in one-cell embryos is due to *de novo* recruitment of maternal MES-4 to incoming sperm chromosomes; this is an interesting contrast to the results above, which demonstrated that both gametes transmit H3K36me3 to embryos.

MES-4 and MET-1 display different dynamics as embryogenesis proceeds. MES-4 is enriched on condensed chromosomes and the levels remain relatively high through the early embryonic divisions, whereas MET-1 is nucleoplasmic and the level of staining diminishes rapidly over the first few embryonic divisions, and then progressively rises after the ∼26-cell stage (Figure 5C). This observation is consistent with previously published data on turn-on of zygotic transcription (Schauer and Wood 1990; Baugh *et al.* 2003; Boeck *et al.* 2016) and our proposed model that maternal MES-4 is a maintenance enzyme for H3K36me3 in early embryos, while MET-1 is likely a transcription-coupled HMT, whose synthesis and subsequent HMT activity depend on activation of the zygotic genome.

### Maternally supplied MES-4 associates with sperm-inherited chromosomes soon after fertilization, and that association depends on their prior marking with H3K36me3

**Figure 6:**
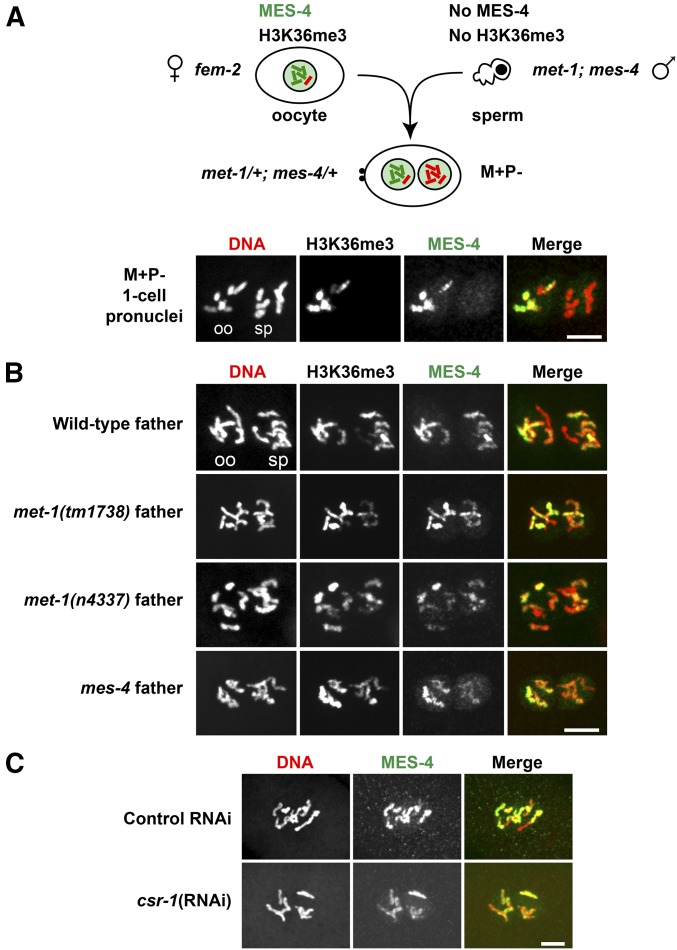
Recruitment of maternally supplied MES-4 to sperm chromosomes requires H3K36me3 generated by either MET-1 or MES-4, and is independent of CSR-1. (A) Immunofluorescence images showing DNA (red), H3K36me3, and MES-4 (green) staining of prometaphase chromosomes in a one-cell M+P− embryo produced by a *fem-2* female mated to a *met-1*(*n4337*); *mes-4*(*bn73*) male. Oocyte-derived (oo) and sperm-derived (sp) chromosomes. Brightness and contrast were enhanced to highlight nucleoplasmic MES-4 in the sperm-derived pronucleus. Maternally supplied chromosomes (M) or paternally supplied chromosomes (P) with H3K36me3 (+) or lacking H3K36me3 (−). Bar, 5 µm. (B) Immunofluorescence images showing DNA (red), H3K36me3, and MES-4 (green) staining of prometaphase chromosomes in one-cell embryos produced by wild-type hermaphrodites, or by *fem-2* females mated to *met-1* or *mes-4*(*bn73*) males. Embryos from wild-type fathers contain paternal chromosomes carrying H3K36me3 generated by MET-1 and MES-4. Embryos from *met-1* fathers contain paternal chromosomes carrying H3K36me3 generated by only MES-4. Embryos from *mes-4* fathers contain paternal chromosomes carrying H3K36me3 generated by only MET-1. Bar, 5 µm. (C) Immunofluorescence images showing DNA (red) and MES-4 (green) staining of prometaphase chromosomes in one-cell embryos produced by RNA interference (RNAi)-treated hermaphrodites. Bar, 5 µm.

Previous chromatin immunoprecipitation studies in *C. elegans* embryos revealed that MES-4 associates with many genes that lack RNA Polymerase II and are not transcribed in embryos but were transcribed in parental germlines (Furuhashi *et al.* 2010; Rechtsteiner *et al.* 2010). This pattern differs from the traditional view that H3K36 HMTs are recruited to genes by elongating RNA Polymerase II. We hypothesized that maternally provided MES-4 is instead recruited to target genes in early embryos by associating with the chromatin modification that it generates, H3K36me3. We tested this possibility by taking advantage of the *de novo* association of maternally provided MES-4 with sperm chromosomes in wild-type one-cell embryos (see section above). If H3K36me3 is required for this *de novo* association, then sperm chromosomes lacking H3K36me3 should fail to recruit maternal MES-4. We mated feminized mothers with *met-1*; *mes-4* fathers to generate M+P− embryos in which the oocyte-contributed chromosomes possessed H3K36me3 and the sperm-contributed chromosomes lacked H3K36me3. We did not detect MES-4 on the sperm-contributed chromosomes, which lacked H3K36me3, whereas MES-4 was highly enriched on the oocyte-contributed chromosomes, which were inherited with H3K36me3 marking (Figure 6A). In these M+P– one-cell embryos, we observed nucleoplasmic MES-4 along with the H3K36me3-negative chromosomes in the sperm pronucleus (Figure 6A), so we can rule out the possibility that maternal MES-4 was not imported into the sperm pronucleus. These findings show that after fertilization, maternal MES-4 is imported into the sperm pronucleus and associates with sperm chromosomes in a manner that requires their prior methylation on H3K36.

We wondered if the source or context of methylation of H3K36 on sperm chromosomes matters for MES-4 recruitment. To test this, we generated one-cell embryos that inherited sperm chromosomes carrying H3K36me3 generated by only MET-1 or only MES-4 by crossing feminized worms to *mes-4* or *met-1* males, respectively. In both cases, MES-4 staining was observed on sperm chromosomes (Figure 6B), indicating that H3K36me3 generated by either HMT is sufficient to recruit maternal MES-4 in one-cell embryos. Since MES-4 is the sole HMT for generating H3K36me2 (Bender *et al.* 2006), these findings also suggest that H3K36me2 is not a critical modification for recruiting maternal MES-4 to chromosomes.

We also considered the possibility that small RNAs play a role in MES-4 recruitment to sperm chromosomes in one-cell embryos, as a growing body of literature implicates small RNAs in transgenerational memory (Stuwe *et al.* 2014). Notably, the genes bound by MES-4 in embryos (4400 genes; Rechtsteiner *et al.* 2010) and the gene targets of the small RNAs bound by the argonaute CSR-1 (4178 genes; Claycomb *et al.* 2009) show significant overlap (3239 genes, *P* < 10^−300^ using a hypergeometric test). To test the possibility that MES-4 recruitment to sperm chromosomes involves CSR-1 or its associated small RNAs, we used RNAi to deplete CSR-1 from maternal germlines and early embryos. Successful RNAi against CSR-1 was confirmed by observation of enlarged and mislocalized P granules in one- and two-cell embryos, as well as lagging chromosomes during anaphase of the first cell division (Claycomb *et al.* 2009; Updike and Strome 2009). The association of MES-4 with chromosomes in one-cell embryos was not altered by depletion of CSR-1 (Figure 6C), suggesting that MES-4 recruitment to chromosomes in one-cell embryos does not require CSR-1.

### MES-4 maintains inherited patterns of H3K36me3 during early embryogenesis

**Figure 7:**
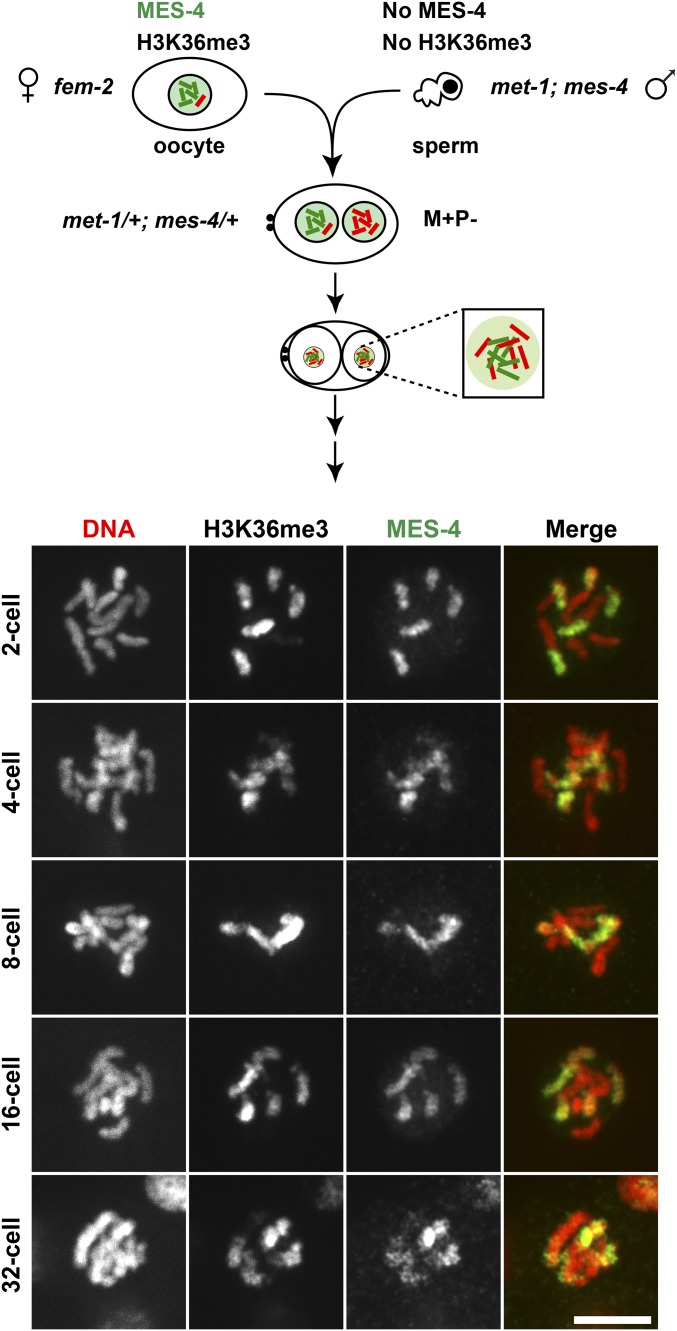
MES-4 maintains H3K36me3 on a subset of chromosomes, likely those that entered the embryo with preexisting H3K36me3. Immunofluorescence images of DNA (red), H3K36me3, and MES-4 (green) on prometaphase chromosomes in single nuclei of M+P− embryos produced by *fem-2* females mated to *met-1*(*n4337*); *mes-4*(*bn73*) males. Maternally supplied chromosomes (M) or paternally supplied chromosomes (P) with H3K36me3 (+) or lacking H3K36me3 (−). Bar, 5 µm.

Differential marking of chromosomes by H3K36me3 and MES-4 in one-cell M+P− embryos provides a unique opportunity to determine if inherited patterns of this histone modification persist, at least at the chromosomal level, through multiple rounds of cell division. If the distributions of histone modifications on chromosomes are transmitted through rounds of DNA replication, we would expect some of the daughter chromosomes to remain marked and some unmarked by H3K36me3 in successive stages of embryogenesis. To test this prediction, we assessed H3K36me3 staining patterns and MES-4 localization in nuclei of M+P− embryos during each prometaphase until the 32-cell stage. The inherited pattern in these embryos was H3K36me3 and MES-4 on oocyte-contributed chromosomes, and not on sperm-contributed chromosomes (Figure 3A). H3K36me3 and MES-4 were maintained on only a subset of chromosomes in each nucleus until the 32-cell stage (Figure 7). After this stage, the nuclei were too small to assess localization of H3K36me3 and MES-4 on individual chromosomes. Because both marked and unmarked chromosomes are present in the same nuclei beginning at the two-cell stage, the maintenance of H3K36me3 and MES-4 on only some chromosomes suggests that the memory of H3K36me3 marking is being maintained only on those chromosomes inherited with H3K36me3. Notably, this maintenance persists until the germline founder cell P4 is born, at the 16–24-cell stage. The germ lineage is the lineage whose survival and development depend on maternal MES-4.

## Discussion

Recent research in the field of epigenetics suggests that gene expression information in the form of histone modifications can be transmitted not only through mitotic cell divisions but also from parents to progeny (Hammoud *et al.* 2009; Furuhashi *et al.* 2010; Rechtsteiner *et al.* 2010; Arico *et al.* 2011; Gaydos *et al.* 2014; Samson *et al.* 2014); this would offer organisms a mechanism to pass a memory of development and life experiences across generations. Major efforts are underway to identify what epigenetic signals are transmitted, which proteins are responsible for generating those signals, how signals are maintained once inherited, and for how many generations signals persist. In this study, we determined that in *C. elegans*: (1) H3K36me3 is transmitted via both sperm and oocyte to progeny, and through cell divisions in the early embryo; (2) two HMTs, MET-1 and MES-4, contribute to H3K36me3 in the germline and in embryos; and (3) maternally supplied MES-4 is responsible for maintaining inherited H3K36me3 in embryos. These data support the previously proposed model (Furuhashi *et al.* 2010; Rechtsteiner *et al.* 2010) that epigenetic information in the form of H3K36me3 is transmitted across generations. This epigenetic information may provide a memory of which genes were expressed in the germ cells in parents and which genes should be turned on in the PGCs of progeny.

The lore in the field is that in organisms with more than one H3K36 HMT, MES-4-related enzymes catalyze H3K36me2 and MET-1-related enzymes catalyze H3K36me3 (Bell *et al.* 2007; Edmunds *et al.* 2008; Wagner and Carpenter 2012). Our previous immunostaining of wild-type and mutant embryos suggested that MES-4 indeed catalyzes all H3K36me2, but that both MET-1 and MES-4 contribute to H3K36me3 (Bender *et al.* 2006; Furuhashi *et al.* 2010; Rechtsteiner *et al.* 2010). That view is supported by two findings in this paper: immunostaining of wild-type and mutant adult germlines, and mass spectrometry analysis of histone modifications in wild-type and mutant embryos. The latter shows that H3K36me3 persists in the absence of MET-1, supporting the existence of at least one additional H3K36me3 HMT. MES-4 is the only other known *C. elegans* H3K36 HMT, and is of particular interest because of its unique ability to maintain methylation of H3K36 in the absence of transcription (Bender *et al.* 2006; Furuhashi *et al.* 2010; Rechtsteiner *et al.* 2010). In the absence of MET-1, H3K36me3 was detected on both canonical H3.1 and variant H3.3, suggesting that MES-4 can methylate histones after both replication-dependent and replication-independent histone incorporation, and can, therefore, propagate a histone-based memory through both transcription- and replication-induced nucleosome disruption (Margueron and Reinberg 2010; Lanzuolo *et al.* 2011; Talbert and Henikoff 2017). The recent discovery that worms lacking H3.3 do not display developmental or fertility defects at normal laboratory temperatures (Delaney *et al.* 2018) suggests that H3K36me3 on H3.3 also does not serve an essential role.

H3K27me3 and H3K9me2/3 are known to be propagated by recognition of the mark by the enzyme complex that makes the mark [PRC2 for H2K27me3 and SU(VAR)3-9 for H3K9me2/3], and subsequent generation of more of the same mark on nearby nucleosomes (Bannister *et al.* 2001; Margueron *et al.* 2009; Xu *et al.* 2010; Poepsel *et al.* 2018). Our study sheds light on the passage and maintenance of H3K36me3, a mark associated with active genes. In *C. elegans*, H3K36me3-marked chromosomes carrying a memory of gene expression from the parental germline are passed from parent to progeny via both sperm and oocyte. Once delivered to the embryo, perpetuation of marked chromosomes through the early embryonic cell divisions relies on MES-4, which is transmitted to the embryo via the oocyte and must newly associate with sperm chromosomes. That association requires that the sperm chromosomes be premarked with H3K36me3. These findings suggest that transmission of H3K36me3 involves MES-4 being recruited (directly or indirectly) to the mark it makes. For a histone mark to provide transgenerational memory, it needs to be established in the parent, transmitted to the progeny through meiosis and gametogenesis, survive postfertilization chromatin remodeling, and, finally, be maintained during embryogenesis until the appropriate cell type is formed. Evidence for all of these steps has been reported for *C. elegans* (Furuhashi *et al.* 2010; Rechtsteiner *et al.* 2010; Gaydos *et al.* 2014; this paper).

Analyses of chromatin in mouse and human sperm suggest that histone modifications that persist through spermatogenesis may influence gene expression in embryos. During spermatogenesis in mice and humans, the majority of histones are replaced with protamines. However, histones and histone modifications are retained at the promoters of some developmentally important loci (Hammoud *et al.* 2009; Jung *et al.* 2017). This packaging in sperm includes active H3K4me3 and repressive H3K27me3 marks, and bivalent marking by both H3K4me3 and H3K27me3 on some genes that are expressed in early embryos, suggesting that at least some histone modifications on sperm chromatin may poise the genome for gene expression during embryogenesis (Hammoud *et al.* 2009; Jung *et al.* 2017). A challenge to that view comes from recent studies reporting that inherited bivalent marking at developmental gene promoters is erased in early embryos and then restored at a later stage (Zheng *et al.* 2016). This conflict highlights the need to study potential mechanisms of epigenetic memory at high temporal resolution and in multiple organisms.

In contrast to the maintenance activity of MES-4, several findings support *C. elegans* MET-1 being a transcription-coupled HMT. MET-1 marks the oocyte X chromosome with H3K36me3 during the late stages of oogenesis, when transcription of X-linked genes is turned on. In embryos, the maternal load of MET-1 is reduced to nearly undetectable levels by the eight-cell stage, and becomes increasingly detectable during early embryogenesis when zygotic transcription also increases. Most transcription-coupled H3K36 HMTs contain a conserved SRI domain that mediates binding of the HMT to the C-terminal tail of elongating RNA Polymerase II. The SRI domain was first described in yeast Set2, and later in *Drosophila* and mammalian homologs of Set2 (Kizer *et al.* 2005; Morris *et al.* 2005; Rebehmed *et al.* 2014). MET-1 contains a sequence with moderate sequence similarity to the SRI domain at a typical position (C-terminal region), while MES-4 contains a sequence with only minimal similarity to an SRI domain at an atypical position (overlapping the SET domain) (B. Strahl, personal communication). Given that MET-1 appears to be primarily responsible for transcription-coupled H3K36me3 and likely is involved in the establishment of an epigenetic memory of gene expression in parental germ cells, it is noteworthy that *met-1* mutants are generally healthy and fertile. MES-4 may well contribute to transcription-coupled H3K36me3 in the germline, although previous results suggest that in embryos it cannot generate H3K36me3 *de novo* but instead is devoted to a maintenance role (Furuhashi *et al.* 2010).

The paradigm of heritable epigenetic repression mediated by *Drosophila* PRC2 and H3K27me3 also includes antagonism, or antirepression, by trithorax group proteins (Klymenko and Müller 2004; Kassis *et al.* 2017). The trithorax group of proteins, which includes an H3K36 HMT, protects genes from PRC2-mediated repression. In worms, MES-4 and methylated H3K36 antagonize deposition of H3K27me3 (Gaydos *et al.* 2012). *In vitro* assays demonstrate that PRC2 is unable to methylate nucleosomes with preexisting H3K36me2 or me3 (Schmitges *et al.* 2011; Yuan *et al.* 2011). Embryos that do not receive maternal MES-4 develop into sterile adults, possibly because the memory of expressed germline genes is not delivered to the PGCs. One likely consequence of losing the memory of gene expression is the encroachment of H3K27me3 and the inappropriate silencing of genes required for the germline developmental program. Indeed, depletion of MES-4 from embryos leads to loss of H3K36me3 from germline genes and the acquisition of H3K27me3 on those genes (Rechtsteiner *et al.* 2010). Therefore, the failure to develop a mature germline in *mes-4* mutants may be the result of inheriting an altered epigenome, silencing of genes required for germline development, and inappropriately expressing genes not normally expressed as part of the germline program.

Maintenance of gene expression patterns is required to ensure that cell fates are maintained. If cells within a tissue lose or change fate, the function of that tissue may be compromised or become cancerous if cells revert to a proliferative state. A memory that is transmitted across generations could influence not only the development of the inheriting organism, but also the fitness of the species as a whole. This is an exciting possibility, and current efforts are focused on determining if environmental factors can change the epigenome, if and how changes are transmitted to progeny, and if such changes influence development in subsequent generations. In *C. elegans*, it is clear that the MES chromatin factors PRC2 and MES-4 function antagonistically across generations to promote germline development. It is likely that the patterns of H3K27me3 and H3K36me3 inherited by the PGCs serve to guide gene expression patterns as they do during *Drosophila* embryogenesis. To test this possibility, ongoing work is aimed at analyzing the gene expression changes in *C. elegans* PGCs that did not inherit MES memory from parent worms.
